# New insights on clinical perspectives of FLASH radiotherapy: from low- to very high electron energy

**DOI:** 10.3389/fonc.2023.1254601

**Published:** 2023-10-23

**Authors:** Stefano Ursino, Giovanni Gadducci, Noemi Giannini, Alessandra Gonnelli, Taiushia Fuentes, Fabio Di Martino, Fabiola Paiar

**Affiliations:** ^1^ Department of Translational Research and New Technologies in Medicine and Surgery, University of Pisa, Pisa, Italy; ^2^ Centro Pisano Multidisciplinare sulla Ricerca e implementazione clinica della Flash Radiotherapy (CPFR), University of Pisa, Pisa, Italy; ^3^ Center for Instrument Sharing University of Pisa (CISUP), University of Pisa, Pisa, Italy; ^4^ Unit of Medical Physics, S. Chiara University Hospital, Pisa, Italy

**Keywords:** FLASH radiotherapy, dose rate, low electron energy, very high electron energy, tumor control probability, normal tissue complication probability

## Abstract

Radiotherapy (RT) is performed in approximately 75% of patients with cancer, and its efficacy is often hampered by the low tolerance of the surrounding normal tissues. Recent advancements have demonstrated the potential to widen the therapeutic window using “very short” radiation treatment delivery (from a conventional dose rate between 0.5 Gy/min and 2 Gy/min to more than 40 Gy/s) causing a significant increase of normal tissue tolerance without varying the tumor effect. This phenomenon is called “FLASH Effect (FE)” and has been discovered by using electrons. Although several physical, dosimetric, and radiobiological aspects need to be clarified, current preclinical “*in vivo*” studies have reported a significant protective effect of FLASH RT on neurocognitive function, skin toxicity, lung fibrosis, and bowel injury. Therefore, the current radiobiological premises lay the foundation for groundbreaking potentials in clinical translation, which could be addressed to an initial application of Low Energy Electron FLASH (LEE) for the treatment of superficial tumors to a subsequent Very High Energy Electron FLASH (VHEE) for the treatment of deep tumors. Herein, we report a clinical investigational scenario that, if supported by preclinical studies, could be drawn in the near future.

## Introduction

The success of radiotherapy (RT) in eradicating tumors depends on the total radiation dose delivered to the tumor. The tolerance of surrounding normal tissues often represents the main limitation in achieving the required dose. In recent decades, technological advances have improved the geometric precision of dose delivery by generating highly conformal dose distributions. However, a significant percentage of tumors remain incurable because of the unacceptable risk-to-benefit ratio (1).

Recently, the possibility of increasing the therapeutic window has been shown through temporal modulation of the beam delivery due to a differential radiobiological effect between normal tissues and tumor, the so called “FLASH Effect” (FE) (2). Specifically, this surprising effect was demonstrated “*in vivo*” on different animal models and organs by delivering the total radiation dose in a very short time (<200 ms), with average dose rates above 40 Gy/s (in comparison with 0.5 Gy/min–20 Gy/min of conventional RT) and leading to a sparing effect on normal tissues without varying tumor effect compared with conventional RT (CONV-RT). As this phenomenon has been discovered using electrons, most preclinical experiments have been performed using this type of radiation source (3–5).

Despite encouraging preclinical findings, the clinical translation of FE is currently in its early experimental phase, primarily owing to significant uncertainties in several key physical, dosimetric, and radiobiological aspects. The design of new devices capable of delivering beam fluences several orders higher than those used for CONV-RT, the availability of new dosimeters, and dosimetric protocols capable of measuring beams at the very high dose per pulse needed to trigger the effect, the dependence of FE on the variations of different temporal beam parameters needs to be clarified before translation to the clinic (6, 7). In addition, a comprehensive understanding of the underlying radiobiological mechanisms driving FE remains to be elucidated.

FE represents a great challenge that could significantly change the paradigm of RT-based treatments in the near future. For this purpose, real brainstorming has been triggered, involving all the specific skills around this issue, from medical physics to biophysics, radiochemists, and radiobiologists to radiation oncologists.

Based on the groundbreaking clinical possibilities associated with FE, we present a detailed overview of the primary oncologic conditions that are particularly suitable for initial clinical investigations of FLASH-RT, from Low Energy Electrons (LEE) (4 MeV–12 MeV) to Very High Energy Electrons (VHEE) (100 MeV–200 MeV) RT.

## Radiobiological premises of clinical translation

The therapeutic window is the dose region interval between the Normal Tissue Complication Probability (NTCP) and the Tumor Control Probability (TCP) curves and represents the milestone for a RT treatment (8). Historically, according to the five principles of radiobiology (5Rs), the use of standard fractionation exploits a differential biological effect favoring the killing of cancer cells more than the killing of healthy ones. Several altered fractionation schemes (such as hyperfractionation or accelerated) have been tested trying to biologically spacing out the two curves but have been gradually abandoned due to controversial risk to benefit ratio (9).

In recent decades, owing to technological advances highly biologically effective hypofractionation schemes have emerged to overcome the intrinsic radioresistance of several tumors, often leading to high curative rates. However, hypofractionation allowed by the geometrical achievement of a very steep spatial dose gradient between the tumor and surrounding healthy tissues remains restricted to limited-sized lesions mostly located in parallel functional organs (such as the lung or liver). Moreover, the use of high linear energy transfer ionizing charged radiation, characterized by a higher relative biological efficacy, has been hampered by the necessity to achieve a subtle balance in the delivery mode to spatially spare the nearest healthy tissues with a subsequent high risk of severe sequelae. These limitations restrict the use of charged particles in clinical practice to very highly selected cases (such as chordoma or non-operable adenoidocystic carcinoma) that must be referred to a few reference centers and often require patients (pts) to be enrolled in clinical trials (10).

Hence, the potential protective effect observed in nonmalignant tissues holds significant promise for novel clinical applications. However, before the translation of FE into clinical practice, several concerns must be addressed through future preclinical studies. First, the determination of the threshold dose per fraction required to trigger the FE, as well as its dependency on various physical beam parameters (such as the total duration of irradiation, average dose rate, dose per pulse, and instantaneous dose per pulse), needs to be resolved. Understanding these dose–response relationships is crucial for optimizing treatment protocols.

Second, the extension of the FE effect to larger irradiation volumes (referred to as the “volume effect”) and exploration of multiple treatment fields are important considerations. Currently, FE has only been observed for a single treatment field delivering a high dose per fraction (approximately 6 Gy) without a standardized set of physical beam parameters, resulting in limited reproducibility of the results (11, 12).

In this regard, we believe that a key point will be to test the sparing effect and the corresponding dose-modifying factor (the increased dose factor to cause the same toxicity grade of CONV-RT) for both organs with “serial” (i.e., spinal cord, small bowel, brainstem and brain tissue) and “parallel” (i.e., lung and liver) functional organization, as well as the isoefficacy on different types of tumors compared with CONV-RT.

Since the FE has already been described as a “tissue effect,” the majority of data to address the clinical translation will likely come from “*in vivo*” experiments. In this regard, the currently available data seem to agree in recognizing a higher tolerance to FLASH irradiation for the most crucial healthy tissues (such as bowel, brain, and lung) that strongly limit the delivery of tumoricidal doses in clinical practice (3–5). Therefore, these results might realistically widen the therapeutic index for oncological conditions that are currently burdened by a dismal prognosis.

## Clinical perspectives

Based on the radiobiological premises reported above, FE could advantageously be translated to the clinic for three main purposes:

1. Radioresistant tumors located in close proximity to radiosensitive “serial” organs: it is hypothesized that a higher RT dose could be delivered to the tumor without inducing severe toxicities to the surrounding normal tissues as would be expected following CONV-RT.
2. Large tumors arising in “parallel” organs: the delivery of tumoricidal RT dose is hampered by the size and local extension of the tumor mass, which would lead to low-dose irradiation of a significant portion of organs at risk with a subsequent unacceptable risk of severe toxicity.
3. Reirradiation: Tumor recurrence often occurs within a previously irradiated high-dose region. This means that the dose required for tumor control is often much higher than that required for severe toxicity, leading to an inverted relationship between NTCP and TCP curves. Currently, these situations are evaluated on a case-by-case basis, taking into consideration the availability of advanced technologies (e.g., Cyberknife) that allow for maximal geometric sparing of primary organs at risk.

From a clinical perspective, two different lines of technological developments can be identified: LEE for the treatment of superficial tumors and VHEE for the treatment of deep tumors.

Undoubtedly, LEE might have very few but, at the same time, rapid clinical applications owing to the ease of technological implementation of LEE accelerators for clinical use. In contrast, VHEE would necessitate the design and development of a novel prototype machine that combines the capacity of managing accelerated high-energy electrons for clinical use with the limited size requirements of an RT bunker. Notably, the latter could be applied to many clinical situations that are currently undercured, thus leading to a real “cutting-edge” breakthrough in the RT treatment of cancer.

## Low electron energy FLASH therapy

Three possible areas of clinical implementation can be identified for LEE: skin tumors, uveal melanoma, and intraoperative RT for abdominopelvic tumors.

Skin tumors usually takes advantage of an upfront surgical removal of the primary lesion despite burdened by a high rate of local recurrence due to their frequent unfavorable location (such a canthus, glabella, nasolabial folds, or preauricolar region) that often limits radical resection causing post-surgical “close” or “microscopically positive” margins (13–15). Therefore, low-energy standard RT (plesiotherapy or superficial brachytherapy) is frequently used as exclusive or postoperative treatment to sterilize microscopic neoplastic foci. Surely, the skin would be the ideal target for a preliminary clinical investigation as it represents a bidimensional matrix that makes it easy for both treatment planning and visualization of tumor and normal tissue response. In addition, the treatment of skin with LEE might play a pioneering role in the subsequent implementation of VHEE, as the healthy skin would represent a primary organ at risk for the treatment of deep tumors.

Uveal melanoma is a rare intraocular tumor that can be treated with radical or conservative treatment (16). Radical treatment, performed in approximately 30% of cases, consists of surgical resection, often providing total removal of the ocular globe and is usually offered to patients with greater tumor size and visual impairment at diagnosis. Indeed, conservative treatment that is performed in approximately 70% of cases consists of RT that can be delivered in the presence of small-sized tumors by implantation of intraocular radioactive plaques (ruthenium or iodine) or in the presence of medium-sized tumors by external beam-accelerated protons (17). The key point in performing conservative treatment is represented by the preservation of the optic nerve, whose damage is related to the reduction or loss of vision (18). Thus, the spatial relationship between tumor and optic nerve is crucial and the choice of external beam protons therapy is based on the achievement of a very steep “fall-off” of RT dose outside the clinical target volume (19). In this regard, owing to the proximity of the target to the cutaneous surface (approximately 1 cm–3 cm), LEE might represent a valid alternative to proton therapy as a conservative treatment in the presence of close proximity or tumor infiltration of the optic nerve. In this regard, preclinical studies investigating the effects of FLASH-RT on peripheral nerves are of primary importance.

Finally, malignancies located in the abdomen or pelvis would optimally fit for an early clinical investigation of Intraoperative Electron FLASH RT (20, 21) Primary tumor control, often conditioning survival due to a higher risk of local mortal complications and the development of distant metastases, can be crucial in the presence of non-operated radioresistant malignancies, such as pancreatic tumors or retroperitoneal sarcoma, surrounded by radiosensitive “serial” organs at risk (such as the intestine or stomach) (22). From the beginning, the rationale for intraoperative RT has always been the improvement of local control through the delivery of a dose-escalated hypofractionated boost to the primary gross tumor after standard fractionated external RT. Nevertheless, late small vessel and peripheral nerve injuries caused by large doses per fraction remains a limiting factor that can only be partially overcome by exploiting intraoperative FE.

Although the therapeutic applications of LEE are limited, it will be the first to be implemented as a direct application of *in vivo* experimental evidence performed using a single field of low-energy electron beams, mechanically collimated, and delivered in a single fraction.

## Very high electron energy FLASH therapy

Multiple brain metastases and primary brain tumors (high grade gliomas) are currently associated to a poor “quoad-vitam” prognosis that is caused by the impossibility to eradicate intracranial tumor disease (23, 24). In fact, the large dissemination of small tumoral foci within the brain (multiple metastases) or the presence of a large tumor mass (primary tumors) usually requires very large-volume irradiation (25). This requirement, combined with the radiosensitivity of healthy brain tissue, usually represents the main limitation for the delivery of high tumoricidal doses (26). Therefore, a whole brain palliative irradiation as well as a “gross tumor” or “tumor surgical bed” involved field with large clinical margins is usually used in the current clinical practice but with suboptimal oncologic results. Although preliminary, currently available preclinical data report promising results in terms of neurocognitive sparing after FLASH compared with conventional irradiation, suggesting a preserving effect on stem hippocampal cells. Specifically, the results of all the cognitive tests performed were statistically indistinguishable between non-irradiated and FLASH irradiated mice, whereas cognition was permanently altered in mice receiving conventional radiation with a dose-modifying factor (DMF) of approximately 1.4 after a single dose of 10 Gy (5). If confirmed, these results could auspiciously pave the way for phase II dose escalation trials aimed at improving the local control of intracranial disease with a subsequent likely improvement in overall survival.

Locally advanced non-small cell lung tumors accounts for approximately 30% of all non-small cell lung tumors (27). Among them, only one-third are suitable for a surgical approach, usually after neoadjuvant chemotherapy. The remaining patients, who are not suitable for surgery, undergo RT-based treatment, either in combination with chemotherapy or as a standalone modality. Although RT represents a fundamental treatment for this subset of tumors, survival is still poor, accounting for approximately 15%–20% of patients alive 5 years after the diagnosis (28). In this case, the dismal prognosis seems to be related to scarce control of the tumor, with a subsequent high risk of life-threatening complications (i.e., hemorrhage) and metastatic dissemination. The real limit is represented by the volume of a healthy uninvolved lung that receives a low radiation dose rather than the maximum dose. In this regard, the life-threatening toxicities that limit the delivery of tumoricidal doses are acute pneumonitis and diffuse late fibrosis (29–31). Again, preclinical “*in vivo*” studies focused on FE in healthy lung tissue reported the occurrence of lung fibrosis after doses much higher than those required with CONV-RT (17 Gy CONV-RT vs. 30 Gy FLASH) (32). These data lay the foundation for a possible and promising translation to the clinic.

Vertebral metastases usually require palliative RT in more than 90% of patients with primary intent to control pain, prevent impending fracture, and avoid intracanal tumor invasion with the subsequent risk of spinal cord compression. Radiation oncologists often fail to reach this goal because of the delivery of suboptimal RT doses. The main drawback is the need to treat the entire vertebra encompassing the spinal cord in the clinical target volume. The spinal cord is a primary radiosensitive “serial” structure and is considered the cornerstone of primary organs at risk in RT, as overcoming the maximum tolerated dose leads to transversal myelitis (33). To date, the preserving effect of FLASH irradiation on the spinal cord has yet to be investigated in preclinical studies, as no data are currently available. Indeed, we do believe that this specific issue should be considered in the planning of the upcoming “*in vivo*” studies as it could rapidly open new perspectives in the clinical translation of FLASH-RT. In fact, the achievement of the maximum tolerated dose in the spinal cord is a major limitation in the RT of vertebral metastases as well as in the reirradiation of locoregional recurrences such as head and neck or lung tumors (34–36). Notably, RT for vertebral metastases might represent an ideal initial clinical application of VHEE due to the necessity of a low-complexity treatment technique.

Finally, pancreatic cancers are notoriously associated with a dismal prognosis due to poor local control and the early occurrence of distant metastases (37). Surgery is the mainstay of treatment, but only 30% of patients are fit, so many of them are treated with chemotherapy or RT. In this regard, the role of RT (mostly in non-surgical patients) has historically been debated because high tumoricidal doses cannot be delivered owing to double limitations. First, in patients affected by tumors located in the head of the pancreas, the duodenum is in close proximity with the primary tumor. Second, the presence of the small bowel, colon, and stomach, surrounding the clinical target volume. Notably, studies focused on investigating the possible role of stereotactic ablative RT in pancreatic cancers failed to prove a clinical benefit owing to the high pattern of severe complications (38) despite the use of a steep-gradient dose delivery technique. Therefore, if the current available data on the preservation of the intestine after FLASH irradiation is proven, it will constitute a good foundation for the potential clinical application of VHEE in pancreatic patients.

The clinical impact of VHEE is extremely important in oncology. However, its clinical implementation requires technological problems to be solved and the radiobiological mechanisms to be properly examined. To date, a clinical VHEE Linac has yet to be implemented. In this regard, high-energy electrons cannot be mechanically collimated (such as low-energy electrons) so that the dose coverage of the irradiated volume can be obtained by using a pencil beam delivery mode. Thus, it is necessary to investigate the “volume effect” by using adjacent and/or overlapping fields. Finally, owing to the necessity of using multiple fields to obtain an acceptable pattern of dose distribution, it is crucial to understand if the time lapse to pass from one field to another could compromise the FE.

## Conclusions

Currently, FLASH-RT has generated a significant interest in the radiation oncology community. Since the inception of RT and its underlying radiobiological principles, it represents the first radiobiological breakthrough that has the potential to revolutionize the treatment paradigm in the field of oncology. It is worth noting that the American Society for Radiation Oncology (ASTRO) membership has acknowledged FLASH-RT as a groundbreaking discovery that warrants prompt translation into clinical practice (39). In this regard, we anticipate an initial phase of limited clinical application involving Low Energy Electron (LEE) radiation, which will hopefully pave the way for a broader clinical implementation of Very High Energy Electron (VHEE) radiation. We believe that the crucial milestones for the exclusive treatment of deep-seated tumors will depend on the sustained manifestation of the FLASH Effect (FE) using low-dose fractions and the application of large, multi-field irradiation techniques. Conversely, FLASH-RT can also be integrated with conventional RT (CONV-RT) in the form of a tumor hypofractionated boost using simple techniques.

## Data availability statement

The original contributions presented in the study are included in the article/supplementary material. Further inquiries can be directed to the corresponding author.

## Author contributions

SU: Conceptualization, Writing – original draft. GG: Writing – review & editing. NG: Writing – review & editing. AG: Writing – review & editing. TF: Writing – review & editing. FD: Conceptualization, Supervision, Validation, Writing – review & editing. FP: Conceptualization, Supervision, Validation, Writing – review & editing.
